# Prevalence of COPD and its association with socioeconomic status in China: Findings from China Chronic Disease Risk Factor Surveillance 2007

**DOI:** 10.1186/1471-2458-11-586

**Published:** 2011-07-22

**Authors:** Peng Yin, Mei Zhang, Yichong Li, Yong Jiang, Wenhua Zhao

**Affiliations:** 1 National Center for Chronic and Noncommunicable Disease Control and Prevention, Chinese Center for Disease Control and Prevention, 27 Nanwei Road, Xicheng District, Beijing, China

## Abstract

**Background:**

Socioeconomic status is likely an independent risk factor for Chronic Obstructive Pulmonary Disease (COPD), but little research has been done in China to study this association in a nationwide sample.

**Methods:**

We used data from the 2007 China Chronic Disease Risk Factor Surveillance of 49,363 Chinese men and women aged 15-69 years to examine the association between the prevalence of self-reported physician diagnosed COPD and socioeconomic status defined by both educational level and annual household income. Multivariable logistic regression modelling was performed with adjustement for potential confounders.

**Results:**

Both low educational attainment and low household income were independently associated with higher risk of physician-diagnosed COPD. Compared to subjects with high educational level, subjects with low educational level had a significantly increased risk of COPD (OR 1.67, 95%CI 1.32-2.13, p for trend< 0.001 for urban, OR 1.76, 95%CI 1.34-2.30, p for trend < 0.001 for rural) after adjusting for age, sex, smoking status, passive smoking and geographic regions. Similarly increased risk was observed for household income and COPD in urban (OR 1.64, 95%CI 1.28-2.09, P for trend< 0.001) but not rural areas. Among never smokers, low educational level and household income were still associated with a significant higher prevalence of COPD (OR 1.77, 95%CI 1.40-2.25, OR 1.31, 95%CI 1.05-1.62). Removal of those with asthma diagnosis did not alter the observed associations.

**Conclusions:**

Socioeconomic status is a risk factor for self-reported physician-diagnosed COPD independently of current or passive smoking. Prospective studies are needed in China to better understand the association between socioeconomic status and COPD.

## Background

Chronic Obstructive Pulmonary Disease (COPD) is a major cause of chronic morbidity and mortality throughout the world [1]. COPD mortality has been rising steadily over the past few decades and it is projected to be the third leading cause of death in the world by 2020 [2]. Cigarette smoking is the major risk factor for COPD; passive smoking, indoor air pollution, occupational dust exposure and genetic factors are recognized as potential factors contributing to the development of COPD [1,3].

There have been many prevalence studies of COPD across countries and regions. A recent meta-analysis [4] comprehensively summarized all the 62 papers published from 28 different countries during 1990-2004 and estimated the pooled prevalence of COPD was 6.4%, 1.8% and 9.2% according to the definition of chronic bronchitis, emphysema and airflow obstruction respectively. Compared to the information available from developed countries, studies of COPD prevalence in China are limited. Three studies done in China were included in Halbert's review [4], one used physician diagnosed emphysema [5] and the other two [6,7] used respiratory symptoms as the case definition. A study in Nanjing, east China, reported that the overall prevalence of physician-diagnosed COPD, using self-reported data from over 29,000 adults over the age of 35 years, was 5.9% [8]. Another study in Guangdong Province, using spirometry to define COPD, reported 9.4% prevalence based on 3286 subjects aged over 40 from two localities. The most recently published population based prevalence study was part of the worldwide Burden of Lung Disease (BOLD study) [9]. They used Global Initiative for Chronic Obstructive Lung Disease (GOLD) criteria to define COPD and recruited more than 20,000 subjects aged 40 years and older from 7 provinces and cities including urban and rural areas from China. The COPD prevalence was highest in Chongqing (13.7%) and lowest in Shanghai (3.9%) among urban areas, and highest in Guangdong (12.0%) and lowest in Liaoning (6.8%) and Shanxi (6.9%) among rural areas. There is no prevalence study carried out in a nationwide sample in China.

Many studies from developed countries suggested that socioeconomic status (SES), measured by income and educational level, is associated with lung function and COPD in terms of exacerbation, prevalence and mortality [10-13]. This association may be partly explained by the greater proportion of smokers among people in lower socioeconomic groups, but smoking may not explain all of the association. In the Chinese population, COPD is prevalent not only among smokers but also among never smokers. This study aimed to clarify this association.

We used nationwide data from China Chronic Disease Risk Factor Surveillance in 2007 to estimate the prevalence of self-reported physician diagnosed COPD and to examine its association with SES.

## Methods

### Study population

**Figure 1:**
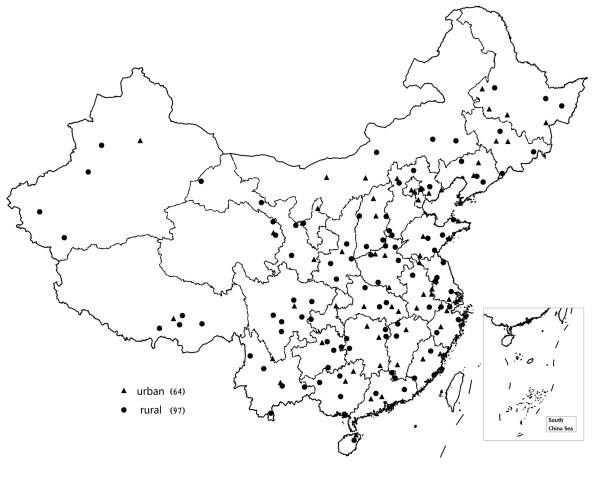
**Geographic distribution of Disease Surveillance Points (DSP) system**.

The field survey of China Chronic Disease Risk Factor Surveillance was carried out from August to October in 2007 based on the China National Disease Surveillance Points (DSP) system. The DSP system included 161 counties/districts (97 counties in rural areas and 64 districts in urban areas) from 31 provinces, autonomous regions and municipalities covering a population of 73 million in China (Figure 1). The system has been shown to be representative of the whole population in China and detailed descriptions of the system were published elsewhere [14].

**Figure 2:**
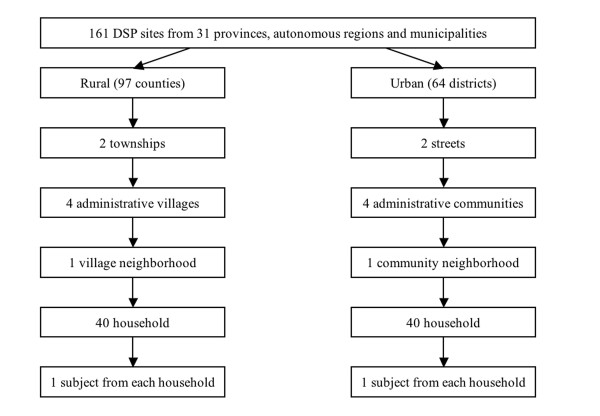
**Flow chart of multi-stage sampling procedure of China Chronic Disease Risk Factor Surveillance 2007**.

Multi-stage cluster sampling was used to select a representative sample of residents aged 15-69 years living in sample areas (Figure 2). Using proportional probability sampling (PPS) method, 2 townships (in rural areas) or streets (urban areas) were selected from each DSP site; also using PPS, 4 administrative villages/communities were selected from each sampled township/street; 1 village neighborhood/communicty neighhorbood was selected from each village/community by simple random sampling. In every selected village/communicty neighhorbood, at least 40 households were selected by simple random sampling and 1 subject was determined by KISH grid method from the selected household. A total of 51,520 subjects were sampled in the surveillance.

Households of similar structure in terms of family member and socioeconomic class were used as replacement if interviewers could not reach the selected family after three attempts. The overall replacement rate was 9.4%.

All recruited residents were invited to participate in the survey at a convenient and accessible site or at home. A standardized questionnaire was administerd by trained interviewers during a face to face individual interview. Data collected in the questionnaire included demographic information, lifestyle factors related to chronic diseases such as smoking, alcohol consumption, physical activity, diet and personal and family medical history. Physical measurements were also taken on all subjects including height, weight, waist circumference and blood pressure.

Of 51,520 subjects sampled from 31 provinces, autonomous regions and municipalities, 49,363 subjects with complete data were included in the analysis in this report. The survey received ethics approval from the Ethics Committee of the Chinese Center for Disease Control and Prevention. All participants gave written, informed consent before participating in the survey.

### Measurements of socioeconomic status and COPD

Educational level and household income were used as indicators for SES. Subjects with education ≥ 12 years, 9-11 years and < 9 years were classified as high, middle and low educational level. Annual household income was classified in high, middle and low level according to tertiles (> 20,000, 8,400-20,000 and < 8,400 RMB). COPD was defined by self-reported physician diagnosis. Subjects who answered the following question affirmatively were classified as having COPD: 'Do you have chronic bronchitis or emphysema or COPD diagnosed by a health professional at township hospital or community hospital or higher hospital?'. The township hospital (in rural) and community hospital (in urban) were equivalent to Grade 1 hospital. In China, hospitals are classified by grade, from higher to lower: grade 3, grade 2, grade 1 and village clinics/community health station.

### Other measurements

Subjects from 31 provinces were classified into eastern (8), central (11) and western (12) depending on geographic distribution according to China National Bureau of Statistics. Each geographic group included urban areas and rural areas. Smoking status was determined by asking 'do you currently smoke cigarettes or pipes? '. Subjects who answered 'yes' were classfied as current smokers. Non current smokers were further asked 'did you smoke in the past?' those who answered 'yes' were defined as ex-smokers. Subjects who answered 'no' in both questions were defined as never smokers. Subjects who were exposed to smoke exhaled by a smoker for more than 15 minutes a day and more than once per week were classified to have passive smoking exposure.

### Statistical analysis

Statistical analyses were performed with SPSS 17.0 for windows (Chicago, IL). COPD prevalence was calculated as crude rates and 95% confidence intervals (CIs) and standardized for age using the world standard population 2000. Differences in COPD prevalence between variables were determined by chi-square tests. Logistic regression was applied to examine the relationship between indicators of socioeconomic status and COPD, from which odds ratio and 95% confidence interval (95%CI) were computed. Test for trend was calculated in the same logistic regression model using educational level and annual household income as continuous variables. The model was adjusted for potential confounders which included age, sex, smoking status, geographic region, and passive smoking exposure. Stratified analyses were performed by smoking status (never and ever smokers) and age groups (30-69 years). To avoid potential misclassification of COPD, we repeated the analysis by excluding those with self-reported asthma diagnosis.

## Results

### Study subjects

Out of 49,363 subjects, there were 23,218 males and 26,145 females, 19,328 in urban areas and 30,035 in rural areas. Subjects were nearly equally distributed in eastern, western and central areas in China. Characteristics of the study population stratified by education and income are shown in table 1. A greater proportion of subjects reported lower educational level in rural than in urban (54.3% vs 27.1%). This pattern was also observed for household income. Subjects in western China tended to have lower educational level and lower household income than central and eastern China. The ever smoking rate was 67.5% in men and 3.6% in women. More than half of men (51.6%) and 41.8% of women were exposed to passive smoking.

**Table 1:** Socioeconomic status of subjects by age, sex, geographic regions and urban/rural areas

		Educational level			Household income		
		
Characteristics	Total n	≥ 12 years	9-11 years	< 9 years	High	Middle	Low
Total	49363	11449 (23.2)	16465 (33.4)	21449 (43.5)	13210 (29.9)	16366 (37.1)	14545 (33.0)
Age							
15-29	7929	2504 (31.6)	3629 (45.8)	1796 (22.7)	2323 (33.2)	2722 (38.9)	1949 (27.9)
30-39	11536	3000 (26.0)	4420 (38.3)	4116 (35.7)	3357 (32.0)	4130 (39.3)	3019 (28.7)
40-49	12133	3294 (27.1)	4313 (35.5)	4526 (37.3)	3184 (29.1)	4414 (40.4)	3329 (30.5)
50-59	11067	1749 (15.8)	2890 (26.1)	6428 (58.1)	2754 (27.9)	3463 (35.1)	3639 (36.9)
60-69	6698	902 (13.5)	1213 (18.1)	4583 (68.4)	1592 (27.3)	1637 (28.0)	2609 (44.7)
Sex							
Men	23218	5885 (25.3)	8668 (37.3)	8665 (37.3)	6203 (29.7)	7711 (37.0)	6937 (33.3)
Women	26145	5564 (21.3)	7797 (29.8)	12784 (48.9)	7007 (30.1)	8655 (37.2)	7608 (32.7)
Urban/rural							
Urban	19328	7567 (39.2)	6531 (33.8)	5230 (27.1)	8226 (47.8)	5917 (34.4)	3066 (17.8)
Rural	30035	3882 (12.9)	9934 (33.1)	16219 (54.0)	4984 (18.5)	10449 (38.8)	11479 (42.7)
Geographic region							
West	17184	2956 (17.2)	5143 (29.9)	9085 (52.9)	2885 (18.9)	5224 (34.2)	7176 (46.9)
Central	15427	3877 (25.1)	5362 (34.8)	6188 (40.1)	3760 (27.4)	5797 (42.3)	4160 (30.3)
East	16752	4616 (27.6)	5960 (35.6)	6176 (36.9)	6565 (43.4)	5345 (35.4)	3209 (21.2)
Smoking status							
Never	32529	7758 (23.8)	10275 (31.6)	14496 (44.6)	9003 (31.2)	10575 (36.6)	9319 (32.2)
Ever	16503	3638 (22.0)	6077 (36.8)	6788 (41.2)	4136 (27.7)	5693 (38.1)	5104 (34.2)
Passive smoking							
Yes	22770	5579 (24.5)	7726 (33.9)	9465 (41.6)	6408 (31.3)	7790 (38.0)	6304 (30.7)
No	26308	5786 (22.0)	8618 (32.8)	11904 (45.2)	6702 (28.7)	8499 (36.4)	8160 (34.9)

### Prevalence of COPD

The overall crude prevalence of COPD was 2.9% (95%CI 2.8-3.1) based on self-report. Standardized to world standard population 2000, the prevalence rate of COPD was 2.2% (95%CI 2.1-2.4%). Among 1423 subjects with COPD, 53.1% were never smokers.

The prevalence among men was significantly higher than among women (3.4% vs. 2.4%, p < 0.001), rural area higher than urban area (3.1% vs. 2.5%, p < 0.001), and western areas (3.7%) higher than central (2.7%) and eastern (2.2%) areas (table 2). The prevalence increased with age, from 0.8% in the youngest age group (15-29 years) to 7.5% in the eldest age group (60-69 years). Former smokers had a higher prevalence of COPD (9.0%) compared to current smokers (3.3%) and never smokers (2.3%). Those with educational level < 9 years had a higher COPD prevalence than subjects with educational level ≥ 12 years (4.2% vs. 1.6%, p < 0.001). Similarly, subjects with low household income had a higher prevalence of COPD compared with those with high household income (4.1% vs. 2.2%, p < 0.001).

**Table 2:** Prevalence of physician-diagnosed COPD by geographic areas in China

	Urban			Rural			Total			
		
	Subjects	COPD	Prevalence (95%CI)	Subjects	COPD	Prevalence (95%CI)	Subjects	COPD	Prevalence (95%CI)	P value*
West	4290	135	3.1 (2.6-3.6)	12894	509	3.9 (3.6-4.2)	17184	644	3.7 (3.4-4.0)	0.019
Central	6840	200	2.9 (2.5-3.3)	8587	211	2.5 (2.2-2.8)	15427	411	2.7 (2.4-3.0)	0.078
East	8198	149	1.8 (1.5-2.1)	8554	219	2.6 (2.3-2.9)	16752	368	2.2 (2.0-2.4)	0.001
Total	19328	484	2.5 (2.3-2.7)	30035	939	3.1 (2.9-3.3)	49363	1423	2.9 (2.8-3.1)	< 0.001

*Comparison between urban and rural with COPD prevalence

### SES and COPD

Educational level and household income were associated with significantly higher prevalence of COPD in both urban and rural areas. As shown in table 3, after adjusting for age, sex, smoking status, passive smoking and geographic regions, the odds for those with < 9 years educational level were 1.67 (1.32-2.13) in urban and 1.76 (1.34-2.30) in rural areas compared with subjects with education ≥ 12 years. The trend was significant in crude and adjusted model. Compared to subjects with high household income, those with low household income had a higher risk of COPD prevalence (OR 1.64, 95%CI 1.28-2.09) in urban areas. Low household income was associated with high COPD prevalence in rural areas in crude analysis, but not after adjustment for potential confounders (p for trend = 0.466).

**Table 3:** Association (crude and adjusted odds ratio and 95%CI) between socioeconomic status and COPD prevalence in urban and rural*

		Urban			Rural	
	
	COPD (%)	Crude OR	Adjusted OR	COPD (%)	Crude OR	Adjusted OR
Educational level						
≥ 12 years	118 (1.6)	1	1	66 (1.7)	1	1
9-11 years	146 (2.2)	1.44 (1.13-1.84)	1.23 (0.96-1.57)	190 (1.9)	1.13 (0.85-1.50)	1.21 (0.90-1.61)
< 9 years	220 (4.2)	2.77 (2.21-3.48)	1.67 (1.32-2.13)	683 (4.2)	2.54 (1.97-3.28)	1.76 (1.34-2.30)
P for trend		< 0.001	< 0.001		< 0.001	< 0.001
Income						
High	165 (2.0)	1	1	132 (2.6)	1	1
Middle	144 (2.4)	1.22 (0.97-1.53)	1.15 (0.91-1.45)	257 (2.5)	0.93 (0.75-1.15)	0.84 (0.68-1.04)
Low	126 (4.1)	2.09 (1.65-2.65)	1.64 (1.28-2.09)	465 (4.1)	1.55 (1.28-1.89)	1.03 (0.84-1.26)
P for trend		< 0.001	< 0.001		< 0.001	0.279

*Adjusted for age, sex, smoking status, passive smoking exposure and geographic regions.

When stratified by smoking status, there was still a significant association between educational level and prevalence of COPD in both smokers (OR 1.64, 95%CI 1.26-2.12 ) and never smokers(OR 1.77, 95%CI 1.40-2.25), suggesting that educational level is a risk factor for COPD independent of smoking (table 4). The association between household income and COPD was observed in never smokers, but not in smokers. Due to the potential change in educational attainment and income between 15-30 years, we repeated the analysis in a subgroup of subjects aged 30-69 years and found similar trend and pattern observed among all study subjects. Exclusion of those with asthma diagnosis did not alter the observed associations (table 5).

**Table 4:** Adjusted odds ratio and 95% CI of SES on COPD prevalence according to smoking status*

		Never smoker		Smoker
	**COPD (%)**	**Adjusted OR(95%CI)**	**COPD (%)**	**Adjusted OR(95%CI)**

Educational level				
≥ 12 years	101 (1.3)	1	80 (2.2)	1
9-11 years	142 (1.4)	1.06 (0.81-1.37)	191 (3.1)	1.36 (1.04-1.78)
< 9 years	509 (3.5)	1.77 (1.40-2.25)	392 (5.8)	1.64 (1.26-2.12)
P for trend		< 0.001		< 0.001
Household Income				
High	162 (1.8)	1	132 (3.2)	1
Middle	213 (2.0)	1.08 (0.87-1.34)	186 (3.3)	0.88 (0.69-1.11)
Low	305 (3.3)	1.31 (1.05-1.62)	284 (5.6)	1.17 (0.93-1.48)
P for trend		0.011		0.071

*Adjusted for age, sex, passive smoking exposure and geographic regions

**Table 5:** Adjusted odds ratio and 95%CI of SES for COPD among different subgroups *

	Aged 30-69 years		Excluding subjects with asthma	
	**Urban**	**Rural**	**Urban**	**Rural**

Educational level				
≥ 12 years	1	1	1	1
9-11 years	1.17 (0.91-1.51)	1.14 (0.84-1.53)	1.12 (0.86-1.46)	1.07 (0.79-1.46)
< 9 years	1.62 (1.27-2.06)	1.61 (1.23-2.12)	1.59 (1.24-2.05)	1.60 (1.21-2.12)
P for trend	< 0.001	< 0.001	< 0.001	< 0.001
Income				
High	1	1	1	1
Middle	1.12 (0.88-1.42)	0.86 (0.69-1.08)	1.05 (0.82-1.35)	0.78 (0.62-0.98)
Low	1.60 (1.25-2.01)	1.01 (0.82-1.25)	1.52 (1.17-1.97)	0.91 (0.73-1.14)
P for trend	< 0.001	0.466	< 0.001	0.942

*Adjusted for age, sex, smoking status, passive smoking exposure and geographic regions

## Discussion

To our knowledge, this is the first study conducted in a nationwide sample covering all 31 provinces, municipalities and autonomous regions of China to estimate the prevalence of COPD based on self-report. We found the prevalence of COPD among resident aged 15-69 years was 2.9%, with geographic variation from 2.2% in the east to 2.7% in the central and 3.7% in the west. Prevalence increased with age, and was significantly associated with smoking and rural areas, consistent with other studies [8,9].

There have been a number of studies in China that reported prevalence of COPD either in certain provinces or selected combination of provinces which cannot provide information on variation of COPD prevalence. Zhong et al reported COPD prevalence in 11 provinces, and found the highest prevalence in Chongqing (west) and lowest in Shanghai (east) among urban areas and highest in Guangdong (east) and lowest in Liaoning (east) among rural areas [9]. Our findings agree with previous reports that the prevalence of COPD was highest in western areas and lowest in eastern areas. This can be explained by the different economic levels in these regions in China. Solid fuel use for cooking and heating in the household without adequate ventilation in west China might contribute to the high prevalence of COPD in the areas. Poorer nutrition, greater occupational exposure and lower health care standard may also play an important part in the significantly higher COPD prevalence in western China compared to central and eastern China. The geographic variation of COPD prevalence in China we found in the present study is consistent with other studies from Latin American and developed countries [15,16].

We found a significant relationship between socioeconomic status reflected by educational level and household income and COPD prevalence in both urban and rural areas. Low household income was associated with high prevalence of COPD in urban areas. This is in accordance with previous studies [8,9,17]. In rural areas, we found no association between income and COPD after adjusting for confounding factors. This might add to the evidence that houshold income is not a good indicator for SES in rural China as self-reported income may be missclassified whether intentionally or unintentionally in rural Chinese pupulation.

Smoking has been well documented as a major cause of COPD, yet never smokers comprise a substantial proportion of individuals with COPD [18] and previous studies indicated that the prevalence of COPD in never smokers is high in China [19]. It is important to note that the dose response relationship between educational level and COPD were found in both never smokers and smokers, suggesting educational level might be a risk factor for COPD independent of smoking. Possible mechanism explaining the adverse effects of low SES on COPD among never smokers might be poor dietary habits (low in antioxidants and fresh fruit) [20,21], poor housing conditions [22], more occupational dust exposure and indoor air pollution from biomass combustion in low SES group [23].

Educational level and household income are two imperfect indicators for socioeconomic status. Highest educational level is usually achieved in young adulthood and income earning generally begin after education is completed. The subjects included in our sample were aged 15-69 years and some of them have not achieved the highest educational level yet. Thus educational level may not reflect changes in social status that occur after schooling ends. The same association we found in the subgroup aged 30-69 years confirmed the dose response association between educational level and COPD. As features of asthma can overlap with COPD to some extent, we repeated the analysis excluding those with self reported asthma to avoid potential misclassification. Exclusion of the subgroup did not alter the magnitude of OR for the association.

We used self reported physician diagnosis as definition to COPD and the reporting relied on level of knowledge of the terms COPD, chronic bronchitis and emphysema in the population. In urban areas, COPD (known as 'Man Zu Fei' in Chinese), chronic bronchitis and emphysema are common terms, especially among the patients. Chronic bronchitis and emphysema are more widely used terms when the doctors announce a diagnosis of COPD in rural areas. The estimation of self-reported COPD prevalence is therefore reliable since it's less likely that the subjects would misreport whether they had physician diagnosed COPD.

The study population included in the analysis represents the largest study estimating COPD prevalence and addressing the relation between socioeconomic status and COPD in China. Subjects were randomly selected from all 31 provinces, autonomous regions and municipalities in China and covered a wide range of age group and variable socioeconomic status. We were able to examine the association in never smokers separately without the major confounding factor of active smoking.

One limitation of the study is its cross-sectional nature. We cannot infer temporal association between SES and COPD. It is difficult to know whether low income is the result of COPD or a risk factor, as the disease may lead to disability and job loss which directly cause decrease of the income. Yet it's unlikely that COPD would alter the educational attainment. Secondly, biomass exposure and occupational dust exposure were implicated as potential risk factors for COPD [3,24,25]. We were not able to adjust for these two factors. Indoor air pollution from biomass combustion is more common in rural areas and we have examined the relation in urban and rural separately. The evidence of adverse effect of occupational dust exposure on COPD is not consistent compared with smoking [26-29], and we have adjusted the main risk factor like smoking and passive smoking. It was therefore unlikely that adding these two factors into the model would have yielded significantly different results. Thirdly, our study population has an upper limit of age of 70 years. The prevalence of COPD is therefore relatively underestimated due to the absence of elderly people who is at highest risk of COPD. The lack of confirmation of COPD diagnosis might also limit the interpretation of the finding due to the difficulty of performing spirometry in this large scale nationwide study. As this study is based on the national surveillance focusing on chronic diseases and risk factors, we did not collect data on respiratory symptoms and treatments which were both useful to confirm COPD diagnosis. Furthermore, due to general lower health care infrastructure and lack of medical knowledge among people in the west area, COPD patients had less frequent clinic visit compared to those in the central and east. The COPD prevalence might be underestimated in the west areas. The current result, however, showed the high prevalence of COPD and the burden it has caused in China. Information on childhood SES was not collected in this study, it would be worthwhile to examine the relationship between childhood SES and COPD because some previous studies have shown that childhood SES played an important role in the development of adult respiratory diseases [11,22,30]. A large study in the UK analyzed data from 1,204,110 people aged over 35 years old from a general practice database and found that risk of developing COPD was strongly associated with adult height (a marker of SES in early life) [31].

## Conclusions

In conclusion, the prevalence of self-reported COPD is high among residents aged 15-69 years in China based on the nationwide sample from 31 provinces, autonomous regions and municipalities. SES was associated with COPD in both urban and rural areas and in both never smokers and smokers. Future research will be needed to better understand the relationship in order to design appropriate interventions to reduce the burden of COPD in China.

## Competing interests

The authors declare that they have no competing interests.

## Authors' contributions

PY and WZ conceived of the study and participated in its design and coordination. PY carried out data analysis and drafted the manuscript. MZ participated in the design of the study and performed data analysis. YL and YJ participated in the study design and helped to draft the manuscript. All authors read and approved the final manuscript.

## Pre-publication history

The pre-publication history for this paper can be accessed here:

http://www.biomedcentral.com/1471-2458/11/586/prepub
